# Validity of self-reported number of teeth and oral health variables

**DOI:** 10.1186/s12903-016-0248-2

**Published:** 2016-07-15

**Authors:** Daisuke Matsui, Toshiro Yamamoto, Masaru Nishigaki, Fumitaro Miyatani, Isao Watanabe, Teruhide Koyama, Etsuko Ozaki, Nagato Kuriyama, Narisato Kanamura, Yoshiyuki Watanabe

**Affiliations:** Department of Epidemiology for Community Health and Medicine, Kyoto Prefectural University of Medicine, Graduate School of Medical Science, 465 Kajii-cho, Kamigyo-ku, Kyoto 602-0841 Japan; Department of Dental Medicine, Kyoto Prefectural University of Medicine, Graduate School of Medical Science, 465 Kajii-cho, Kamigyo-ku, Kyoto 602-0841 Japan

**Keywords:** Self-report, Questionnaire survey, Number of teeth, Validity

## Abstract

**Background:**

Oral condition and number of teeth were investigated by questionnaire in the Japan Multi-Institutional Collaborative Cohort (J-MICC Study). The aim of the present study was to assess the validity of the tooth number measure by comparing the self-reported number of teeth with the number of teeth determined at clinical dental examination.

**Methods:**

A self-administered questionnaire and dental examination were performed by 1275 participants of a company medical examination who requested dental check-up and 377 subjects of the J-MICC study. The validity of the tooth number measure was assessed by comparing the self-reported number of teeth with that determined at clinical examination. Spearman’s rank correlation coefficient was calculated to quantitatively evaluate the validity.

**Results:**

In males, the mean clinically-examined and self-reported numbers of teeth were 26.5 and 24.8 teeth, respectively. In females, the mean clinically-examined and self-reported numbers of teeth were 26.4 and 25.5 teeth, respectively. There was a tendency toward underestimation of the number of natural teeth by self-reporting. A significant correlation was observed between the clinically-examined and self-reported numbers of teeth in total (*ρ* = 0.69) and both males (*ρ* = 0.70) and females (*ρ* = 0.67).

**Conclusions:**

The self-reported oral health variables were valid and reflected clinical status. Further revision of the question on the remaining tooth in the questionnaire improves the validity of self-reported number of teeth.

## Background

The Japan Multi-Institutional Collaborative Cohort (J-MICC) Study is a large cohort study initiated in 2005 to investigate the gene-environment interaction of lifestyle-related diseases, including cancer, in Japan [1]. Several reports suggested that periodontal disease may be a risk factor for lifestyle-related diseases, such as diabetes [2], cardiovascular disease [3], and metabolic syndrome [4]. In addition, some studies have investigated the association between the number of teeth and dementia [5], oral and gastrointestinal cancer [6], cardiovascular mortality [7, 8], and risk of mortality [8]. It is now commonly accepted that dental and oral health is relevant to general systemic health.

Oral health data collected by clinical dental examinations have been considered as the only valid source of information [9]. However the clinical dental examinations have intensive in terms of personnel, facilities, time, and cost. Information gained through questionnaire is alternative sources of data on oral health status. If the self-reported oral health measurement is valid, it would provide a more convenient process for measuring oral health conditions in populations and groups at lower cost, less resource involvement, and within shorter timeframes. Some studies have reported the validity of self-reported measures such as number of teeth and use of dentures [10–13]. But in their study, there were small numbers of subjects and these surveys have been conducted in Europe or America [10, 11]. Moreover, Japanese survey had many numbers of subjects, however the population age was 40–56 years [12]. Douglass CW et al. [13] reported a high correlation coefficient, however the measure was a telephone survey. Oral conditions and number of teeth were investigated by a questionnaire in the J-MICC Study. However, the validity of these results has not been confirmed. It remains unknown whether this validity is similarly applicable to the subjects of the J-MICC Study, due to differences in subject ethnicity and study areas.

The present study aimed to assess the validity of self-reported number of teeth, by comparison with the number of teeth counted at clinical dental examination.

## Methods

### Subjects

Subjects were enrolled from two sources. One was from individuals who underwent a regular company medical checkup in Kyoto. When they visited the medical examination center, the staff confirmed whether the applicant wished to undergo a dental check-up, and we enrolled 1,275 subjects who indicated interest (response rate is unknown). The other source consisted of subjects of the J-MICC Study living in the Kyoto area. We conducted a baseline survey with approximately 6500 inhabitants aged 35 years or higher in Kyoto prefecture, between 2007 and 2013. We enrolled 377 people who participated in the follow-up survey 5 years after the baseline survey. The survey was performed between November 2013 and November 2014. Of the 1652 subjects, 151 did not complete the questionnaire. Thus, 1501 subjects were included in the present study for analysis.

## Questionnaire

The questionnaire was administered prior to dental examination, and included questions on gender, age, number of remaining teeth, and dental health behaviors, including frequency of tooth brushing, frequency of interdental cleaning instrument use, visiting a dental clinic and frequency of dental scaling visit. Regarding the self-reported number of teeth, the following wording was utilized in the questionnaire: ‘How many natural teeth do you have in your mouth? Excluding wisdom teeth, adults have 28 teeth. Tooth implants should not be included in your total count’. No further instructions on how to conduct the self-assessment were provided to the participants.

### Dental examination

Clinical examination of dental status was performed by local general dentists. Subjects sat down facing a dentist, and were examined using a dental mirror and explorer. The number of original teeth and Community Periodontal Index (CPI) were measured during examination. CPI developed by the Oral Health Unit of WHO in 1997. Severity and degree of periodontal diseases in a section of a population are assessed, according to a WHO-recommendation, by the CPI taking as its basis the three features bleeding, dental calculus, and gingival sulcus [14].

### Statistical analysis

The validity of the tooth number measure was assessed by comparing the self-reported number of teeth with the clinically-examined number of teeth. Spearman’s rank correlation coefficient was calculated to quantitatively evaluate the validity. Oral health variables associated with a difference between the self-reported and clinically-examined numbers of teeth were investigated using Spearman’s rank correlation coefficient. Categories included: clinically-examined numbers of teeth (0–19, 20–32), CPI (0, 1–2, 3–4), frequency of tooth brushing (once, twice, 3 or more times), and frequency of dental scaling visit (none, once or twice, 3 or more). All analyses were performed using SPSS Statistics 21 for Windows (SPSS Japan Inc.).

## Results

**Fig. 1:**
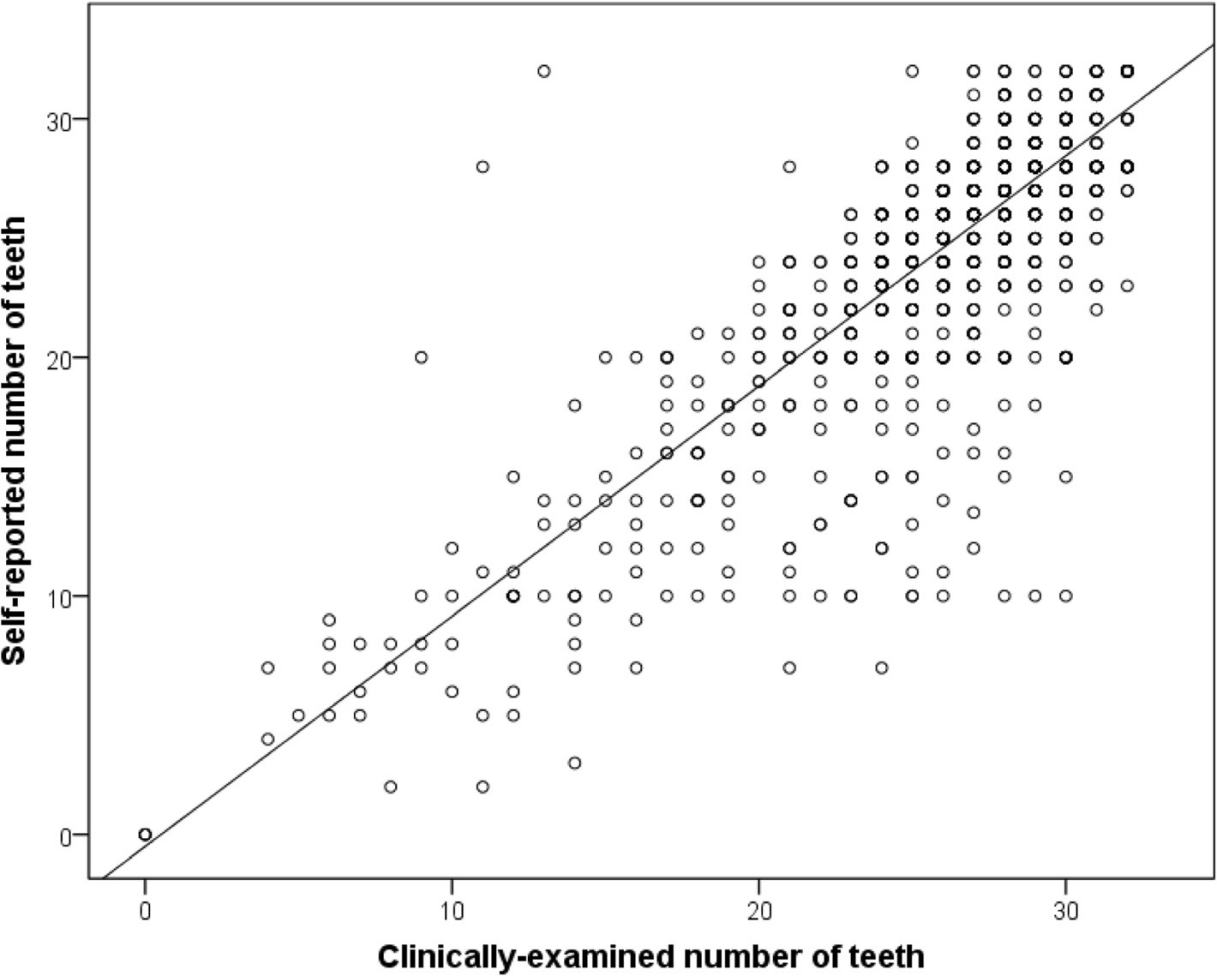
Association of self-reported and clinically-determined numbers of teeth

Table 1 shows the characteristics of the study subjects. In males, the mean (SD) number of clinically-examined teeth [26.5 (4.4)] was significantly higher than the mean (SD) number of self-reported teeth [24.8 (5.3)]. Similarly in females, the mean (SD) number of clinically-examined teeth [26.4 (4.1)] was significantly higher than the mean (SD) number of self-reported teeth [25.5 (4.8)]. Fig. 1 shows a scatter plot of self-reported number of teeth vs clinically-determined number of teeth. The points below the line indicated underestimation of the self-reported number, while the points above the line indicated overestimation. Subjects accurately reported their number of teeth, although there was a slight tendency toward underreporting.

**Table 1:** Subject characteristics

	Male (*n* = 899)	Female (*n* = 612)	Total (*n* = 1501)
Age (mean)	57.7 ± 10.2	55.1 ± 9.6	56.7 ± 10.0
35 ~ 49 years old	224 (25.2 %)	200 (32.7 %)	424 (28.2 %)
50 ~ 59 years old	255 (28.7 %)	208 (34.0 %)	463 (30.8 %)
60 ~ 69 years old	284 (31.9 %)	158 (25.8 %)	442 (29.5 %)
70 ~ 89 years old	126 (14.2 %)	46 (7.5 %)	172 (11.5 %)
Clinically-examined number of teeth	26.5 ± 4.4	26.4 ± 4.1	26.5 ± 4.3
Self-reported number of teeth	24.8 ± 5.3	25.5 ± 4.8	25.1 ± 5.1
Mean of tooth brushing per day	1.9 ± 0.7	2.3 ± 0.7	2.1 ± 0.7
Mean of interdental cleaning instrument use per week	2.5 ± 4.9	3.3 ± 5.0	2.9 ± 4.9
CPI			
0	54 (6.1 %)	91 (14.9 %)	145 (9.7 %)
1,2	608 (68.4 %)	439 (71.7 %)	1047 (69.7 %)
3,4	227 (25.5 %)	79 (12.9 %)	306 (20.4 %)
Not measurable	0	3 (0.5 %)	3 (0.2 %)
Recent visit to dental clinics			
Yes	89 (10.0 %)	64 (10.5 %)	153 (10.2 %)
No	800 (90.0 %)	548 (89.5 %)	1348 (89.8 %)
Frequency of dental scaling visit			
None	453 (51.0 %)	259 (42.3 %)	712 (47.4 %)
Once or twice a year	326 (36.7 %)	265 (43.3 %)	591 (39.4 %)
3 ~ 5 times a year	75 (8.4 %)	72 (11.8 %)	147 (9.8 %)
6 or more times a year	35 (3.9 %)	16 (2.6 %)	51 (3.4 %)

*CPI* community periodontal index

Table 2 shows the comparison between the self-reported and clinically-examined numbers of teeth. A significant correlation was observed between the number of clinically-examined teeth and the number of self-reported teeth in total (*ρ* = 0.69, *p* < 0.01) and both males (*ρ* = 0.70, *p* < 0.01) and females (*ρ* = 0.67, *p* < 0.01). Furthermore, significant correlations between clinically-examined and self-reported teeth number were observed in all age groups, and as age increased, the coefficient of correlation became higher. Table 3 shows the comparison between the self-reported and clinically-examined numbers of teeth in subjects of a regular company medical checkup and J-MICC Study. In subjects of a regular company medical checkup, a significant correlation was observed between the number of clinically-examined teeth and the number of self-reported teeth in total (*ρ* = 0.68, *p* < 0.01). In subjects of J-MICC Study, a significant correlation was observed between the number of clinically-examined teeth and the number of self-reported teeth in total (*ρ* = 0.71, *p* < 0.01).

**Table 2:** Comparison of self-reported and clinically-examined numbers of teeth

	Clinically-examined number of teeth		Self-reported number of teeth		*ρ* ^a^	*p*-value
	min. – max.	mean ± SD	min. – max.	mean ± SD		
Total (1501)	0 - 32	26.5 ± 4.3	0 - 32	25.1 ± 5.1	0.69	<0.01
Male (n)						
35 ~ 49 (224)	22 - 32	28.5 ± 1.8	10 - 32	27.1 ± 2.7	0.43	<0.01
50 ~ 59 (255)	7 - 32	27.7 ± 2.6	8 - 32	26.4 ± 3.5	0.63	<0.01
60 ~ 69 (284)	4 - 32	24.9 ± 5.5	4 - 32	22.8 ± 6.3	0.78	<0.01
70 ~ 87 (126)	4 - 32	24.2 ± 5.7	3 - 32	21.9 ± 6.6	0.82	<0.01
Total (889)	4 - 32	26.5 ± 4.4	3 - 32	24.8 ± 5.3	0.70	<0.01
Female (n)						
35 ~ 49 (200)	11 - 32	27.9 ± 2.3	14 - 32	27.3 ± 2.3	0.47	<0.01
50 ~ 59 (208)	13 - 32	27.4 ± 2.5	10 - 32	26.3 ± 3.6	0.52	<0.01
60 ~ 69 (158)	0 - 31	24.5 ± 5.3	0 - 32	23.2 ± 6.0	0.77	<0.01
70 ~ 89 (46)	6 - 28	22.4 ± 6.2	2 - 28	21.5 ± 7.4	0.91	<0.01
Total (612)	0 - 32	26.4 ± 4.1	0 - 32	25.5 ± 4.8	0.67	<0.01

*min* minimum, *max* maximum, *SD* standard deviation

^a^Spearman’s rank correlation coefficient

**Table 3:** Comparison of self-reported and clinically-examined numbers of teeth in subjects of a regular company medical checkup and J-MICC Study

	Clinically-examined number of teeth		Self-reported number of teeth		*ρ* ^a^	*p*-value
	min. – max.	mean ± SD	min. – max.	mean ± SD		
Subjects 1						
Total (1315)	4 - 32	26.6 ± 4.2	2 - 32	25.2 ± 5.1	0.68	<0.01
Male (n)						
35 ~ 49 (204)	22 - 32	28.5 ± 1.8	10 - 32	27.1 ± 2.7	0.45	<0.01
50 ~ 59 (231)	7 - 32	27.8 ± 2.5	8 - 32	26.6 ± 3.4	0.62	<0.01
60 ~ 69 (249)	5 - 32	25.1 ± 5.6	5 - 32	23.0 ± 6.5	0.77	<0.01
70 ~ 87 (125)	4 - 32	24.1 ± 5.7	3 - 32	21.8 ± 6.6	0.82	<0.01
Total (809)	4 - 32	26.6 ± 4.5	3 - 32	24.9 ± 5.4	0.70	<0.01
Female (n)						
35 ~ 49 (168)	11 - 32	28.0 ± 2.2	14 - 32	27.3 ± 2.2	0.46	<0.01
50 ~ 59 (161)	13 - 32	27.7 ± 2.2	10 - 32	26.8 ± 3.1	0.48	<0.01
60 ~ 69 (133)	7 - 31	24.7 ± 4.3	5 - 32	23.7 ± 5.1	0.78	<0.01
70 ~ 89 (44)	6 - 28	22.4 ± 6.3	2 - 28	21.5 ± 7.6	0.91	<0.01
Total (506)	6 - 32	26.6 ± 3.9	2 - 32	25.7 ± 4.5	0.67	<0.01
Subjects 2						
Total (186)	0 - 32	26.0 ± 4.8	0 - 32	24.3 ± 5.5	0.71	<0.01
Male (n)						
35 ~ 49 (20)	23 - 32	29.0 ± 2.1	20 - 32	27.4 ± 2.7	0.29	0.21
50 ~ 59 (24)	19 - 32	26.7 ± 3.2	15 - 29	24.5 ± 4.0	0.67	<0.01
60 ~ 69 (35)	4 - 29	23.8 ± 4.7	4 - 29	21.7 ± 5.0	0.64	<0.01
70 ~ 87 (1)	30	30	28	28	-	-
Total (80)	4 - 32	26.1 ± 4.3	4 - 32	24.0 ± 4.7	0.73	<0.01
Female (n)						
35 ~ 49 (32)	15 - 32	27.6 ± 2.9	15 - 32	27.2 ± 2.9	0.57	<0.01
50 ~ 59 (47)	14 - 32	26.5 ± 3.0	10 - 30	24.6 ± 4.6	0.57	<0.01
60 ~ 69 (25)	0 - 30	23.0 ± 8.9	0 - 29	20.8 ± 9.1	0.71	<0.01
70 ~ 89 (2)	20 - 23	21.5 ± 2.1	21 - 21	21.0 ± 0	-	-
Total (106)	0 - 32	25.9 ± 5.3	0 - 32	24.4 ± 6.0	0.70	<0.01

Subjects 1: Subjects of a regular company medical checkup

Subjects 2: Subjects of J-MCC Study

*min* minimum, *max* maximum, SD standard deviation

^a^Spearman’s rank correlation coefficient

Table 4 shows the comparison of self-reported and clinically-examined numbers of teeth by oral condition and oral hygiene. A significant correlation was observed between the clinically-examined number of teeth and self-reported number of teeth for the clinically-examined number of teeth, CPI, frequency of tooth brushing and frequency of dental scaling visit in males and females. Table 5 shows the comparison of self-reported and clinically-examined numbers of teeth by oral condition and oral hygiene in subjects of a regular company medical checkup. A significant correlation was observed between the clinically-examined number of teeth and self-reported number of teeth for the clinically-examined number of teeth, CPI, frequency of tooth brushing and frequency of dental scaling visit in males and females. Table 6 shows the comparison of self-reported and clinically-examined numbers of teeth by oral condition and oral hygiene in subjects of J-MICC Study. A significant correlation was observed between the clinically-examined number of teeth and self-reported number of teeth for frequency of dental scaling visit in males and females.

**Table 4:** Comparison of self-reported and clinically-examined numbers of teeth by oral condition and oral hygiene

	Male (*n* = 889)			Female (*n* = 612)		
	n	*ρ* ^a^	*p*-value	n	*ρ* ^a^	*p*-value
Oral condition						
Clinically-examined number of teeth						
0 ~ 19	70	0.72	<0.01	31	0.65	<0.01
20 ~ 32	819	0.63	<0.01	581	0.64	<0.01
CPI						
0	54	0.62	<0.01	91	0.62	<0.01
1,2	608	0.68	<0.01	439	0.65	<0.01
3,4	227	0.78	<0.01	79	0.75	<0.01
Oral hygiene						
Frequency of tooth brushing						
Once a day	249	0.71	<0.01	55	0.69	<0.01
Twice a day	480	0.69	<0.01	312	0.69	<0.01
3 or more times a day	160	0.74	<0.01	245	0.65	<0.01
Frequency of dental scaling visit						
None	453	0.69	<0.01	259	0.74	<0.01
Once or twice a year	326	0.66	<0.01	265	0.58	<0.01
3 or more times a year	110	0.80	<0.01	88	0.65	<0.01

^a^Spearman’s rank correlation coefficient

*CPI* community periodontal index

**Table 5:** Comparison of self-reported and clinically-examined numbers of teeth by oral condition and oral hygiene in subjects of a regular company medical checkup

	Male (*n* = 809)			Female (*n* = 506)		
	n	*ρ* ^a^	*p*-value	n	*ρ* ^a^	*p*-value
Oral condition						
Clinically-examined number of teeth						
0 ~ 19	64	0.70	<0.01	26	0.54	<0.01
20 ~ 32	745	0.61	<0.01	480	0.63	<0.01
CPI						
0	34	0.72	<0.01	48	0.69	<0.01
1,2	557	0.66	<0.01	384	0.63	<0.01
3,4	218	0.78	<0.01	74	0.75	<0.01
Oral hygiene						
Frequency of tooth brushing						
Once a day	230	0.69	<0.01	41	0.74	<0.01
Twice a day	436	0.68	<0.01	263	0.68	<0.01
3 or more times a day	143	0.76	<0.01	202	0.62	<0.01
Frequency of dental scaling visit						
None	404	0.67	<0.01	215	0.74	<0.01
Once or twice a year	306	0.67	<0.01	224	0.54	<0.01
3 or more times a year	99	0.80	<0.01	67	0.71	<0.01

^a^Spearman’s rank correlation coefficient

*CPI* community periodontal index

**Table 6:** Comparison of self-reported and clinically-examined numbers of teeth by oral condition and oral hygiene in subjects of J-MICC Study

	Male (*n* = 80)			Female (*n* = 106)		
	n	*ρ* ^a^	*p*-value	n	*ρ* ^a^	*p*-value
Oral condition						
Clinically-examined number of teeth						
0 ~ 19	6	0.22	0.67	5	1.00	-
20 ~ 32	74	0.68	<0.01	101	0.65	<0.01
CPI						
0	20	0.57	<0.01	43	0.55	<0.01
1,2	51	0.75	<0.01	55	0.74	<0.01
3,4	9	0.55	0.12	5	0.50	0.39
Oral hygiene						
Frequency of tooth brushing						
Once a day	19	0.76	<0.01	14	0.44	0.11
Twice a day	44	0.72	<0.01	49	0.70	<0.01
3 or more times a day	17	0.66	<0.01	43	0.75	<0.01
Frequency of dental scaling visit						
None	49	0.76	<0.01	44	0.75	<0.01
Once or twice a year	20	0.54	0.01	41	0.70	<0.01
3 or more times a year	11	0.89	<0.01	21	0.45	0.04

^a^Spearman’s rank correlation coefficient

*CPI* community periodontal index

## Discussion

The clinically-examined number of teeth significantly correlated with the self-reported number of teeth in both males (*ρ* = 0.70) and females (*ρ* = 0.67) of the present study. Thus, the present results suggested that the self-reported number of teeth is a valid reflection of the clinically-examined number of teeth. Our results showed a lower correlation coefficient than those previously reported [10–13]. A previous study of 40- to 56-year-old Japanese subjects reported a correlation coefficient of 0.80 [12], and a study comprised of 50 subjects older than 70 years reported a correlation coefficient of 0.97 [13]. However, the latter study was a telephone survey, and had directed subjects to count their teeth with a mirror. In contrast, our subjects have high proportion of younger population who might not be careful for the oral health behavior than those previous reports. It may cause that our results indicated lower correlation coefficient.

The present results are consistent with previous studies reporting that the self-reported number of teeth is often lower than that determined during clinically-examined [11, 12]. This may be due to the use of the phrasing “natural teeth,” which may lead patients to not count teeth abutting a crown and bridge. The discrepancy between the number of self-reported and clinically-examined teeth was greatest in those with many prosthetic teeth (data not shown). Therefore, the validity of self-reported number of teeth may be improved if the wording of the questionnaire explicitly explained the characteristics of restorative and prosthodontic dental work, to allow subjects to have a better understanding of their restoration status.

Buhlin et al. [10] reported that older individuals show more concern for their oral health. Moreover, the average number of remaining teeth in the present study was higher than that of the average Japanese population [15], which may be possibly attributed to the subjects’ increased awareness of their oral health. Therefore, the correlation coefficient may increase with age.

Ueno et al. [12] reported that the correlation coefficient of patients with 1–19 teeth is higher than that of patients with 20–32 teeth. This result is in accordance with our result. Also, our results showed significantly negative correlation between CPI and the clinically-determined number of teeth (*r* = -0.13, data not shown). Therefore, the correlation coefficient may increase with CPI.

Some previous studies reported frequency of tooth brushing as a factor associated with the number of remaining teeth [16, 17]. These studies included the nonuse of tooth brushing; however our study did not assess this. Therefore, the subjects’ concern for their oral health may be more related to the choice of brushing their teeth or not, rather than the frequency of tooth brushing.

Nakayama et al. [16] reported that the association between the number of remaining teeth and regular dental check-ups was not significant. Therefore, the association between the frequency of dental scaling and awareness of oral health is not significant, and may have no effect on the correlation between the numbers of remaining and self-reported teeth.

One limitation of the present study is population bias; those voluntarily undergoing medical examination tend to be more conscious about their health. Furthermore, the present study included a higher proportion of both　subjects of a regular company medical checkup and J-MICC Study having 20–32 teeth compared with the Japanese national average [15]. Future studies including more patients with 0–19 teeth are warranted. However, our subjects were superior to previous reports that there were wide age bracket and great numbers of subjects.

## Conclusions

Our results suggested the validity of self-reported number of teeth, and that this measure reflected the number of clinically-examined number of teeth. Further revision of the question on the remaining tooth in the questionnaire improves the validity of self-reported number of teeth.

## Abbreviations

CPI, Community Periodontal Index; SD: standard deviation; J-MICC, Japan Multi-Institutional Collaborative Cohort.
